# Investigation of the Shape-Memory Properties of 3D Printed PLA Structures with Different Infills †

**DOI:** 10.3390/polym13010164

**Published:** 2021-01-05

**Authors:** Guido Ehrmann, Andrea Ehrmann

**Affiliations:** 1 Virtual Institute of Applied Research on Advanced Materials (VIARAM); guido.ehrmann@gmx.de; 2 Faculty of Engineering and Mathematics, Bielefeld University of Applied Sciences, 33619 Bielefeld, Germany

**Keywords:** polylactic acid (PLA), fused deposition modeling, 3-point bending test, infill parameters, infill density, shape-memory properties

## Abstract

Polylactic acid (PLA) belongs to the few thermoplastic polymers that are derived from renewable resources such as corn starch or sugar cane. PLA is often used in 3D printing by fused deposition modeling (FDM) as it is relatively easy to print, does not show warping and can be printed without a closed building chamber. On the other hand, PLA has interesting mechanical properties which are influenced by the printing parameters and geometries. Here we present shape-memory properties of PLA cubes with different infill patterns and percentages, extending the research reported before in a conference paper. We investigate the material response under defined quasi-static load as well as the possibility to restore the original 3D printed shape. The quasi-static flexural properties are linked to the porosity and the infill structure of the samples under investigation as well as to the numbers of closed top layers, examined optically and by simulations. Our results underline the importance of designing the infill patterns carefully to develop samples with desired mechanical properties.

## 1. Introduction

Three-dimensional (3D) printing belongs to the emerging technologies of our time. Especially low-cost printers, mostly based on the fused deposition modeling (FDM) technique, are nowadays used by many people, from private people to the maker community to small companies [1]. Poly(lactic acid) (PLA) belongs to the most often used polymers in 3D printing by the FDM technology. Its biocompatibility enables using it for 3D printed external medical aids [2], in tissue engineering or bone repair [3,4,5]. Diverse conductive and other nanofillers can be introduced into PLA filaments, such as graphene, carbon black or carbon nanotubes, to increase their mechanical and/or conductive properties or to produce 3D printed sensors [6,7]. On the other hand, it is possible to introduce continuous filaments from diverse natural or man-made materials [8,9]. Generally, it is even possible to re-use solid PLA waste from diverse applications, such as food packaging and car dashboards, although at the price of a reduced tensile stress and flexural strength [10].

The mechanical properties of the most often used polymers, such as PLA or acrylonitrile butadiene styrene (ABS), and the surface waviness are often the factors limiting possible applications [11,12,13,14]. Several research groups thus aim at increasing the mechanical properties, e.g., by embedding nanoparticles, nanofibers, microfibers, chemical or heat treatment [15,16,17]. Alternatively, PLA or also flexible FDM polymers can be used to create sandwiches with other objects, e.g., with textile fabrics which can be used to increase the elastic modulus [18,19,20].

Another approach is suggested by some research groups who utilize a special property of PLA, its shape memory [21,22,23], such as Senatov et al. who investigated PLA blended with hydroxyapatite to FDM print porous scaffolds which could be strongly mechanically deformed, resulting in cracking of parts of the scaffolds, and afterwards brought back to the original shape in a self-healing process [22]. Liu et al. blended PLA with SiC and carbon and investigated the recovery rate and recovery time of corresponding filaments and printed specimens. They found the first to be correlated with the thermal conductivity, while a higher amount of SiC resulted in higher recovery forces [23]. Wu et al. investigated the impact of diverse printing parameters on the recovery of FDM printed PLA samples and found the recovery temperature to be of high importance, with 70 °C showing optimum results [24]. It should be mentioned that PLA is not the only 3D printable shape memory polymer (SMP). Other SMPs which can be 3D printed are, e.g., polyurethane [25,26], poly(ε-caprolactone) [27], poly(vinyl alcohol) [28] or poly cyclooctene [29].

While many of the aforementioned papers concentrate on blending PLA with different materials or filling the filament with nanoparticles to add a more rigid phase as a backbone, here we use pure PLA and vary the infill pattern of the test samples instead. Similar to the first studies of Senatov et al. [21,22], we perform mechanically destructive tests and investigate the recovery potential. The research shown here is an extension of the results published in [30].

## 2. Materials and Methods

A 3D printer I3 MK3 (Prusa Research A.S., Prague, Czech) with nozzle diameter 0.4 mm was used to print the specimens at a layer thickness of 0.15 mm and a first layer height of 0.2 mm. The nozzle temperature was set to 210 °C (215 °C for the first layer), and the bed temperature was 60 °C during the whole printing process. To examine the impact of the infill patterns alone, no contours were printed so that the whole specimen consisted of usually 5 compact layers on top (varying between 0 and 10 for one part of the study), 5 compact bottom layers and pure infill between, with overall dimensions of 20 mm × 20 mm × 20 mm.

**Figure 1:**
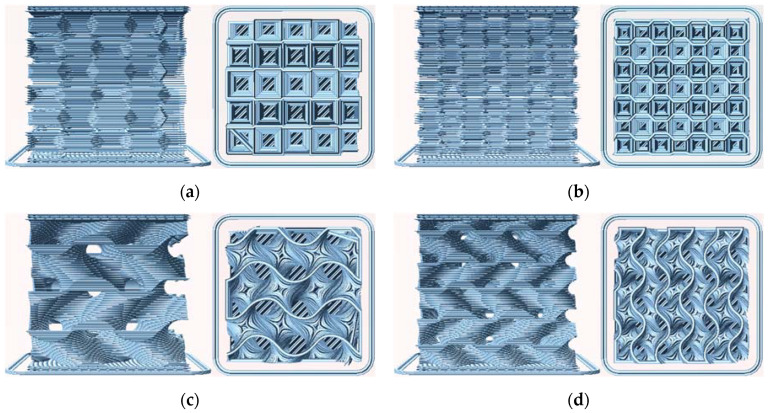
Front (**left**) and top view (**right**) on the samples under examination: (**a**) 3D honeycomb, 10% infill; (**b**) 3D honeycomb, 15% infill; (**c**) gyroid, 10% infill; (**d**) gyroid, 15% infill.

As infill patterns, “gyroid” and “3D honeycomb” were chosen, applying 10% and 15% infill density in case of the 3D honeycomb structure and in addition also 20%, 25% and 30% for the gyroid structure. Some of the specimens are depicted in Figure 1, each from front as well as from top with a cut above layer 50. Depending on the infill pattern and density, the samples are referred to as G (gyroid) or H (honeycomb), e.g., H10 (Figure 1a), H15 (Figure 1b), G10 (Figure 1c) and G15 (Figure 1d). In addition, the number of top layers was varied between 0 and 10 for the gyroid sample with 15% infill, referred to as G15/0 in case of 0 top layers, etc.

Besides the clearly different wall orientations of the chosen infill structures, Figure 1 also shows that the gyroid structure (Figure 1c,d) has channels crossing the whole specimen, which is not the case for the honeycomb structure.

**Figure 2:**
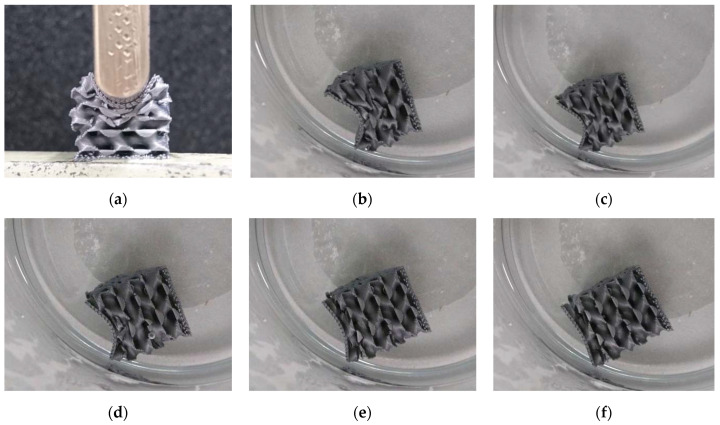
Deformation and recovery processes: (**a**) Pressing sample G15 up to a maximum impact of 10 mm; (**b**–**f**) recovery process in warm water.

Investigations of the mechanical properties were performed in a Sauter TVM-N universal testing machine (Kern & Sohn GmbH, Balingen-Frommern, Germany), combining the single load pin of a 3-point bending test with an even counterpart (cf. Figure 2a). In this way, it was possible to test a local impact on the specimen, opposite to previous studies using two flat planes pressing the sample from opposite sides. Quasi-static load tests were stopped at a force of 1700 N or at a penetration depth of 10 mm, i.e., half the overall sample height, whatever happened first. The tests were performed with a speed of 6 mm/min.

Microscopic images of the samples were taken using a digital microscope Camcolms2 (Velleman, Gavere, Belgium).

Recovery was accomplished after each test inside a water bath which was held at (60 ± 2) °C for 1 min, directly followed by the subsequent mechanical test. It should be mentioned that recovery temperature and duration are generally assumed to be of high importance for the grade of recovery [24]. The focus of our study, however, was on the occurrence of breaks in the structures under investigation which are equivalent to irreversible damage. Thus, in this first investigation of the influence of different infill patterns and percentages, only one parameter set was tested.

Depending on the visual inspection of the sample after recovery, 5 or 10 test cycles were carried out.

## 3. Results and Discussion

Generally, the testing process was performed as depicted in Figure 2, showing sample G15 as an example. Firstly, the sample was deformed in the testing machine (Figure 2a). Directly afterwards, the deformed sample was inserted in warm water for recovery (Figure 2b–f).

**Figure 3:**
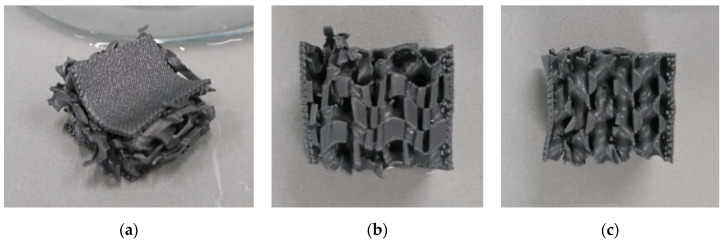
The 10th deformation and recovery processes of sample G15: (**a**) after maximum deformation; (**b**,**c**) after recovery in warm water; images taken from different sides.

This process, however, did not work infinitely long. In each testing cycle, the cracking of single connections which broke under the load was clearly recognizable, indicating that more and more parts of the sample were destroyed. As an example, Figure 3 depicts sample G15 after 10 deformation cycles and the subsequent—incomplete—recovery which leaves back several clearly visible destroyed bonds and deformations which cannot be restored anymore.

**Figure 4:**
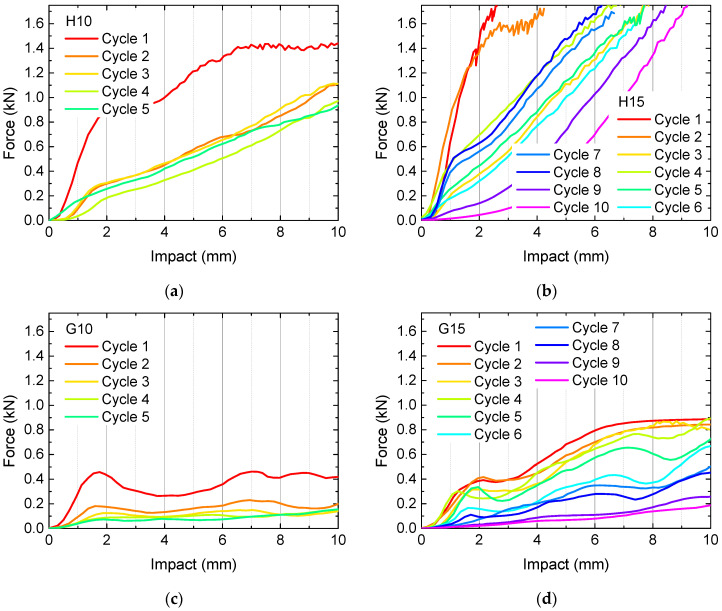
Quasi-static load tests in original state and after several test and recovery processes: (**a**) 3D honeycomb, 10% infill; (**b**) 3D honeycomb, 15% infill; (**c**) gyroid, 10% infill; (**d**) gyroid, 15% infill.

To investigate this behavior quantitatively, quasi-static load tests were performed. The results of samples H10, H15, G10 and G15 are depicted in Figure 4. Measurements were stopped after 5 (samples H10, G10) or 10 cycles (samples H15, G15), respectively, depending on the optical appearance, i.e., when clear breaks throughout nearly whole layers became visible.

Firstly, Figure 4 clearly shows that the samples with 10% infill need significantly lower loads for identical impacts than their counterparts with 15% infill, as expected. Besides, the 3D honeycomb samples show significantly higher loads at identical impact than the gyroid samples. The latter finding can be explained by the structures, depicted in Figure 1. Only the 3D honeycomb samples have vertical walls which can fully counteract the applied forces, while the inner walls in the gyroid samples are always tilted, in this way being easier bendable without the necessity to break directly, enabling taking up a certain load by changing the tilting angles of the walls before the material finally breaks at weak points.

What is also visualized here is the fact that recovery is never complete. Under the conditions chosen here, i.e., recovery at (60 ± 2) °C without waiting times before the next test, the forces at identical impact decrease from one cycle to the next in nearly all cases. Deviations can be attributed to small shifts in the position of the sample between subsequent tests. Comparing with Wu et al. [24], the next tests should be carried out at a higher temperature of 70 °C minimum to possibly enable a higher amount of recovery.

Another important point must be stated regarding the 3D honeycomb samples. As mentioned before, this structure does not have channels through which the water in the water bath can flow. This means, on the other hand, that heat transfer in these samples is incomplete, and the inner structures will recover less than the outer ones. Especially for the denser sample H15, this meant that after 10 test cycles, the imprint of the load pin was still visible after recovery, in this way clearly showing that recovery was incomplete.

**Figure 5:**
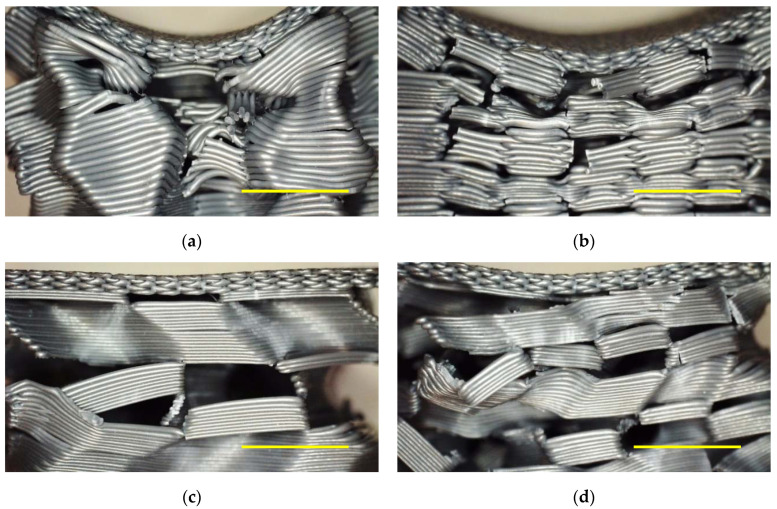
Microscopic images after 5/10 quasi-static load test cycles: (**a**) 3D honeycomb, 10% infill; (**b**) 3D honeycomb, 15% infill; (**c**) gyroid, 10% infill; (**d**) gyroid, 15% infill. Scale bars show 5 mm.

Figure 5 depicts microscopic images of the samples evaluated in Figure 4 after the last recovery cycle. All of them show clearly broken areas, partly correlated with large deviations of sample parts from their original positions. Although only a smaller impact is possible in the 3D honeycomb samples, the residual deformation of the upper layers is larger for them due to the aforementioned incomplete recovery. These images underline that the first samples under examination are not applicable in situations in which constant load changes occur and nearly complete recovery after each cycle is necessary.

**Figure 6:**
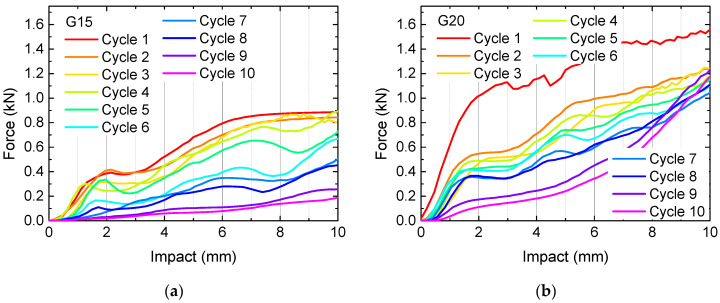

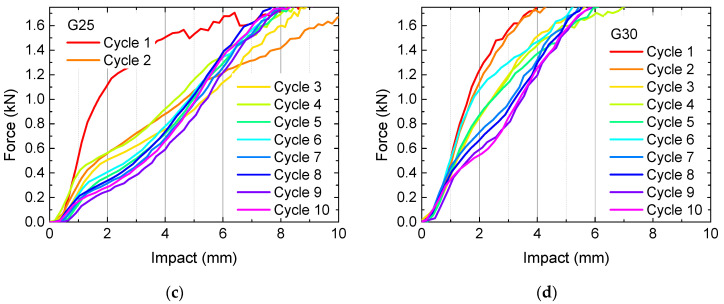
Quasi-static load tests in original state and after several test and recovery processes: gyroid with (**a**) 15% infill; (**b**) 20% infill; (**c**) 25% infill; (**d**) 30% infill.

Since the samples with gyroid infill patterns showed forces well below the maximum values available in the 3-point bending tester and higher recovery properties, as visible from the lower residual bending of the upper layers shown in Figure 5, further tests were performed on gyroid-filled samples with higher infill percentages of 20%, 25%, and 30%. The results are depicted in Figure 6, compared with sample G15 which showed the best properties in the first test cycle.

**Figure 7:**
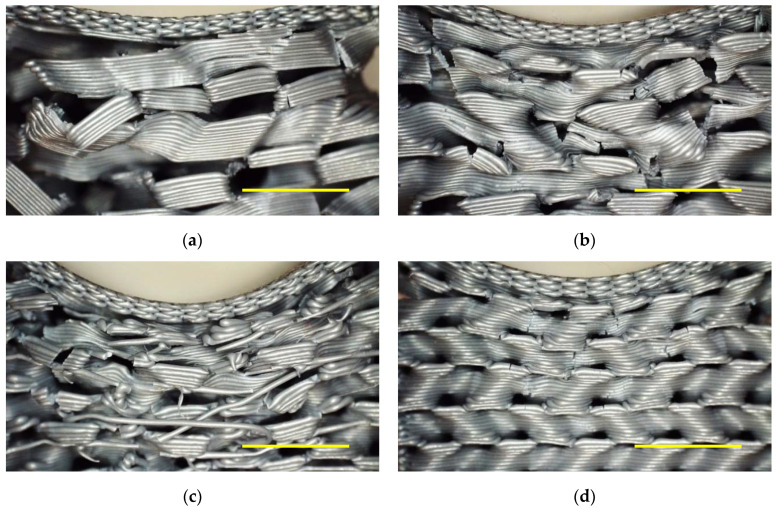
Microscopic images after 10 quasi-static load test cycles: gyroid with (**a**) 15% infill; (**b**) 20% infill; (**c**) 25% infill; (**d**) 30% infill. Scale bars show 5 mm.

While the slopes of the curves generally get steeper with increasing infill percentage, especially for G25 and G30 it can also be recognized that the change of the curves with increasing numbers of tests cycles is much smaller than for the less filled samples. This finding could be interpreted in terms of less broken connections, enabling higher recovery, leading to very similar slopes for each test cycle. However, such an analysis cannot be based purely on the mechanical examinations. Figure 7 thus depicts microscopic images of the samples depicted in Figure 6, taken after 10 testing and recovery cycles.

These images show on the one hand that for an infill of 30% using the gyroid pattern (Figure 7d), there are indeed significantly less broken lines visible than in the other cases. On the other hand, in this case only the upper layers can be influenced at all by the test, while the lower ones seem to be unaltered. This fits well to the curves shown in Figure 6d, indicating that no larger impact than approx. 6 mm could be achieved before the maximum force, defined as 1.7 kN due to the experimental limitations, was reached. On the other hand, these upper layers nevertheless show broken areas, underlining that in spite of the relatively small impact, here nevertheless connections are destroyed during the tests.

The strongest residual deviation from the original shape is clearly visible for an infill of 25% (Figure 7c), as can be seen from the strong bending of the top layers. Here, also many broken and shifted areas are visible. While in many cases such test series result in an optimum value of the test parameter somewhere in the middle of the parameter span, here the opposite seems to be valid—an infill of 25% for the gyroid pattern seems to be the worst decision, combining incompletely absorbing the loads, as visible from Figure 6 where in most cases only an impact of approx. 8 mm could be reached, with nevertheless high amounts of destroyed connections and correspondingly a high residual distortion, i.e., low recovery.

**Figure 8:**
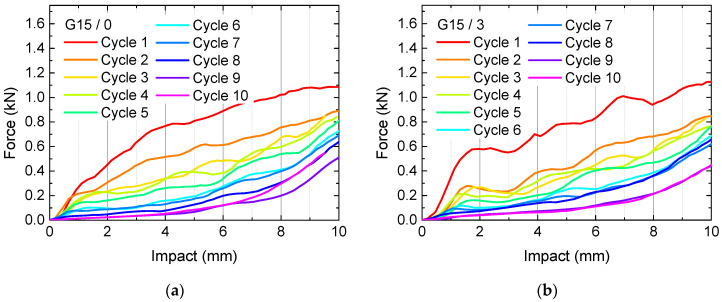

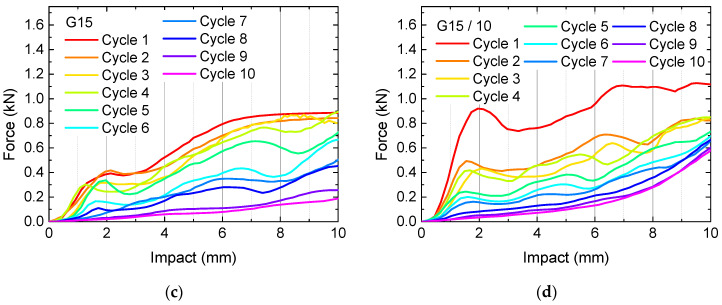
Quasi-static load tests in original state and after several test and recovery processes: gyroid with 15% infill and (**a**) 0 top layers; (**b**) 3 top layers; (**c**) 5 top layers; (**d**) 10 top layers.

**Figure 9:**
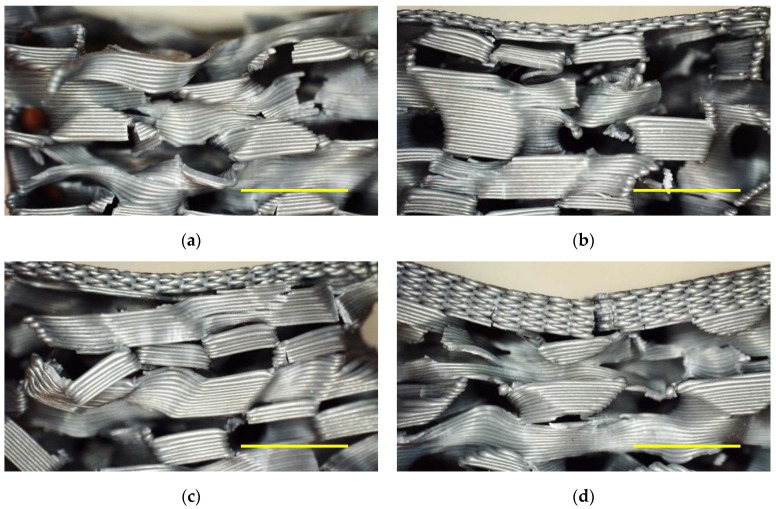
Microscopic images after 10 quasi-static load test cycles: gyroid with 15% infill and (**a**) 0 top layers; (**b**) 3 top layers; (**c**) 5 top layers; (**d**) 10 top layers. Scale bars show 5 mm.

This undesired combination suggests further tests with modified top layers with the aim to spread the load better and thus lead to either better load absorbance, i.e., by using less top layers, or to better spreading the load by using more top layers. Figure 8 and Figure 9 thus show the results of quasi-static load tests and microscopic images of samples with varying numbers of top layers (with a constant number of 5 bottom layers), compared to the reference sample G15 with 5 top layers.

Unexpectedly, here again the average sample G15 apparently shows the smallest forces necessary for an impact of 10 mm after 10 test cycles, indicating that this average value of 5 top layers may be the worst choice. On the other hand, deformations of G15/0 are strongest since there is no top layer keeping the lower layers in shape, while the top layer of sample G15/10 broke during the first test cycles (Figure 9).

Combining these findings, it may be supportive to find a top layer structure which is not fully closed, leading to the relatively constant load dissipation visible in the first cycle of G15/0, and on the other hand still connects the single parts of the infill to avoid large shifts of the material, as it happened for sample G15/0. For this, possibly S-like structures along 0° and 90° may be suitable, or auxetic structures which allow for simultaneous extensions in x- and y-orientation [31].

These first tests of the special infill patterns show that the gyroid filled specimens have a better recovery behavior, but significantly lower load bearing capacities than the 3D honeycomb structure. Regarding the numbers of top layers and the infill percentage, middle values were unexpectedly not ideal, but oppositely showed lowest recovery, indicating that typical optimization strategies do not work here, but new ideas are necessary to prepare samples with higher load dissipation and higher recovery properties. Next, further tests with recovery at higher temperatures and other structures with channels inside the infill, allowing warm water to penetrate into the whole sample, will be tested to optimize load bearing and recovery properties of FDM printed PLA samples.

## 4. Conclusions and Outlook

In a recent study, we investigated the recovery properties of porous PLA specimens due to material’s shape memory properties. Opposite to previous studies, a 3-point bending load pin was used to apply a local load, and new infill patterns—3D honeycomb and gyroid—were tested. While the first structure showed a significantly higher load bearing capacity, the latter had better recovery abilities.

In general, only a certain amount of recovery could be reached, which may be due to the relatively low recovery temperature of 60 °C, especially in case of the 3D honeycomb specimens which did not show continuous channels through which the warm water could penetrate into the inner parts of the samples.

Optimized structures and recovery conditions can be used, e.g., in personal protective equipment, as smart actuators, deployable structures for aerospace [32,33], but also in bone implants [22] or other medical applications.

Future tests will concentrate on varying the recovery temperature and duration as well as the printing temperature and on designing a new structure which combines the advantages of the recent ones, including rotating the infill pattern by 90° via a horizontal axis, equivalent to pressing the recent samples on the side. It is necessary to ensure identical points of load incidence in subsequent experiments to avoid misinterpretations of the results due to slightly shifted sample positions. Finally, using other SMPs, blends of PLA with other SMPs or PLA-soft also have to be investigated as another possibility to increase recovery and reduce the breaks of parts of the structures.

## Data Availability

The data created in this study are fully depicted in the article.
